# The question of WGS’s clinical utility remains unanswered

**DOI:** 10.1038/s41431-021-00823-y

**Published:** 2021-02-17

**Authors:** Florian Battke, Björn Schulte, Martin Schulze, Saskia Biskup

**Affiliations:** 1 CeGaT GmbH, Tübingen, Germany; 2 Praxis für Humangenetik, Tübingen, Germany

**Keywords:** Health care, Social sciences

## To the Editor:

The recent publication by Bertoli-Avella et al. [1], titled “Successful application of genome sequencing in a diagnostic setting: 1007 index cases from a clinically heterogeneous cohort” in your journal provided data on a question that many in the field of human genetics are currently considering: Is it time to move from whole-exome sequencing (WES) to whole-genome sequencing (WGS)? There is a growing body of knowledge about pathogenic variants that may only be accessible to WGS diagnostics. Among those are intronic sequence variants as well as larger structural variants (SVs), both of which remain undetected as the affected regions and breakpoints are not enriched and thus missed in WES. Beyond the currently evaluated variants are more recently discovered events such as disruptions of topologically associated domains.

It is our strong belief that human genetic diagnostics will move from the coding regions to a more comprehensive view of the genome. To find out whether we are already at this point, carefully designed studies are needed that show the clinical benefit of WGS over WES in realistic cohorts. While we appreciate the authors’ efforts in showing this benefit, they ultimately fail to show what they claim.

First, to show the benefit of a new technology such as WGS in clinical practice over the current standard of care, confounding factors must be taken into account and both methods compared fairly. Instead, where the authors do compare both methods directly, the exome data were analyzed some time ago, while the WGS analysis benefits from current knowledge. The authors rightly show that the advancement of medical knowledge improves solution rates, but then go on to claim this as a benefit brought by WGS technology, which it clearly is not.

Second, a large part of the cohort was not analyzed by both technologies. For those cases solved by WGS, the authors do not check whether these cases would have been solved by WES. Instead, they again claim these solutions as a result of WGS technology. As we understand the presented data, 212 cases of 1007 were solved by using WGS, with 41 solved by P/LP variants in non-coding regions while 171 had variants in coding regions. Variants in coding regions are accessible to WES analysis, so we focus on the variants in non-coding regions as those are potential candidates for showing the superiority of WGS. However, 27 of these 41 variants are extremely close to exon borders or expand into coding regions and would certainly not be missed by state-of-the-art WES. Of the 41 solutions due to non-coding variants found by WGS, only 14 can be claimed as showing the benefit of the technology. Interestingly, these 14 variants are all covered in modern exome designs that include known pathogenic and likely pathogenic variants from HGMD and ClinVar and are already used in clinical practice, at least in our institutions.

Third, the most important benefit of genome-wide analysis is the move from sequence variants alone to all kinds of variants including SVs. However, it seems that only one of the considerable number of cases assessed by the authors is solved by detecting structural variation of any kind. Perhaps this cohort really does not include more patients with SVs, or perhaps this is the result of strong filtering needed to reduce the number of false positive SV calls. It is certainly a surprising result and worthy of further discussion.

Taken together, these observations would indicate that the diagnostic yield increase of WGS versus current off-the-shelf WES as shown by the present publication is in the range of 1.4 percentage points (14/1007) and disappears almost completely when using optimized exome kits. As this cohort is partially composed of cases with negative previous WES, it is likely enriched for cases that are particularly hard to solve. In a non-enriched cohort, the increase in diagnostic yield would likely be even smaller.

In summary, the title of the publication is misleading as are the main conclusions. It certainly shows a successful application of WGS in a diagnostic setting, but the main result is that WES would have been a sufficient method to solve the vast majority of cases solved by WGS here—if performed and analyzed according to the state of the art. We fear that the presentation by the authors inflates expectations about WGS solution rates, leading to misconceptions about what is and is not possible with current technologies and, ultimately, to disappointment both of requesting physicians as well as patients.

Advocating that WGS should become the standard-of-care test based on a study that shows hardly any clinical benefit is certainly a hard sell. Given the minimal increased diagnostic yield, the skewed cohort analyzed here, the inability of regular 30× WGS to detect mosaicism, and the relatively poor performance of current SV calling strategies on short-read data, we are not convinced that short-read WGS is the technology that will finally be the comprehensive genetic test the community hopes for.

Nevertheless, we strongly believe that cost efficient long read technologies and deep coverage WGS will be the next important steps in human genetic testing. Until then, properly performed trio whole exome sequencing, including enrichment of known intronic pathogenic and likely pathogenic variants in both parents and the affected individual, with sufficient coverage to also detect mosaicism, and with expert variant assessment should be considered as method of first choice.
